# Assessment of radiation protection awareness and knowledge about radiological examination doses among Italian radiographers

**DOI:** 10.1007/s13244-015-0445-6

**Published:** 2015-11-23

**Authors:** F. Paolicchi, F. Miniati, L. Bastiani, L. Faggioni, A. Ciaramella, I. Creonti, C. Sottocornola, C. Dionisi, D. Caramella

**Affiliations:** Diagnostic and Interventional Radiology, Via Roma 67, 56100 Pisa, Italy; Institute of Clinical Physiology, National Research Council, Via Moruzzi 1, 56124 Pisa, Italy; Department of Medical Physics, Piazzale Ospedale 1, 31100 Treviso, Italy

**Keywords:** Radiation protection, Radiographers, Risk awareness, Radiation dose, Staff training

## Abstract

**Objectives:**

To evaluate radiation protection basic knowledge and dose assessment for radiological procedures among Italian radiographers

**Methods:**

A validated questionnaire was distributed to 780 participants with balanced demographic characteristics and geographic distribution.

**Results:**

Only 12.1 % of participants attended radiation protection courses on a regular basis. Despite 90 % of radiographers stating to have sufficient awareness of radiation protection issues, most of them underestimated the radiation dose of almost all radiological procedures. About 5 % and 4 % of the participants, respectively, claimed that pelvis magnetic resonance imaging and abdominal ultrasound exposed patients to radiation. On the contrary, 7.0 % of the radiographers stated that mammography does not use ionising radiation. About half of participants believed that radiation-induced cancer is not dependent on age or gender and were not able to differentiate between deterministic and stochastic effects. Young radiographers (with less than 3 years of experience) showed a higher level of knowledge compared with the more experienced radiographers.

**Conclusions:**

There is a substantial need for radiographers to improve their awareness of radiation protection issues and their knowledge of radiological procedures. Specific actions such as regular training courses for both undergraduate and postgraduate students as well as for working radiographers must be considered in order to assure patient safety during radiological examinations.

***Main messages*:**

• *Radiographers should improve their knowledge on radiation protection issues.*

• *Only 12.1 % of participants attended radiation protection courses on a regular basis.*

• *Specific actions must be considered in order to increase knowledge and awareness.*

## Introduction

Ionising radiation from medical applications represents the majority of radiation doses from artificial sources to which the general population is exposed. This is the consequence of a steadily increasing demand for radiological examinations with particular reference to multidetector computed tomography (MDCT), which alone accounts for about 50 % of the overall medical radiation exposure [1]. Though this has been paralleled by a dramatic evolution of imaging technology over the last decade, it is often worsened by a lack of appropriateness and optimisation criteria by both referring physicians and radiological staff [2–5]. Recently, efforts by both vendors and societies were carried out to reduce radiation doses and sensitise users and patients to the issues of radiological protection [6, 7]. As shown by several authors, this increasing use of medical radiation can be partly explained by the inaccurate and often inadequate knowledge among professionals about radiation protection issues and radiation doses of commonly performed imaging procedures [8–11]. Such lack of awareness about radiation risk can be extremely dangerous when high dose examinations, such as multiphase MDCT studies, are conducted without optimisation, resulting in a potentially significant biological lifetime risk for patients. The radiation hazard can be particularly relevant for young patients and especially children, whose high biological susceptibility and long life expectancy tend to increase the likelihood of the effects of not only cancer but also other non-cancerous diseases. In this respect, evidence exists that imaging parameters for paediatric examinations are frequently not adjusted to the smaller sizes of children compared with adults, resulting in an unnecessarily high radiation exposure [12–16].

The new Council Directive 2013/59/Euratom of the 5th December 2013, which concerns “laying down basic safety standards for protection against the dangers arising from exposure to ionising radiation”, is poised to strengthen this need for change, imposing on all professionals an ever greater duty of care to properly justify and optimise each radiological procedure [17]. Furthermore, the “Guidelines on radiation protection education and training of medical professionals in the European Union no. 175 (2014)” has set the minimum knowledge expected of each and every practitioner involved in Radiation Protection [18]. These guidelines clearly state the core learning outcomes in radiation protection for radiographers, such as:

To use the appropriate medical devices in an effective, safe and efficient manner

To use effective, safe and efficient radiation protection methods in relation to staff, patients and the general public applying current safety standards, legislation, guidelines and regulations

To apply the concepts and tools for radiation protection optimisation

Information campaigns such as Image Gently, Image Wisely, and the most recent Eurosafe campaign have paid specific attention to the fundamental role of staff training in radiation protection, emphasising the role of strict cooperation among all radiological operators [19, 20]. Inside the radiological team, radiographers play an important role, as they are most directly involved in performing examinations and therefore represent the final gatekeeper in the radiation protection chain.

Our purpose was to perform a cross-sectional survey aimed to assess among Italian radiographers the knowledge of dose exposure levels and awareness of radiation protection issues.

## Material and methods

Data were obtained from a survey issued to Italian radiographers during several educational courses, workshops and meetings held in different Italian regions between 1 January and 31 December 2014. Ethical committee review was not deemed necessary as the survey population did not include any at-risk groups and anonymity was assured to all participants. The questionnaire, which had been validated in advance to perform a prospective observational study, consisted of 22 questions in a multiple choice format and was divided into three sections (Appendix). The various sections were focused on assessing:

1. The demographic features of the participants (i.e. age, title, city/region and formal education in radiation protection) (Section 1).
2. the awareness about radiation protection issues, specifically: (1) standards about radiation, (2) susceptibility to radiation damage, (3) regulations, (4) knowledge about professionals with a higher exposure risk, (5) tissues more susceptible to injury from ionising radiation, (6) diseases caused by radiation damage and (7) knowledge about dose optimisation (Section 2).
3. The knowledge about radiation dose levels of the natural background and common imaging procedures, based either on or without the usage of ionising radiation, and specifically: (1) average dose of a postero-anterior chest X-ray (considered as a common reference unit to compare radiation exposure from different radiological examinations); (2) background radiation dose received by the general population; (3) lumbar spine X-ray dose; (4) mammography dose (bilateral, two projections for each side); (5) chest computed tomography dose; (6) pelvic magnetic resonance dose; (7) positron emission tomography-computed tomography dose; (8) abdominal ultrasound dose; (9) myocardial scintigraphy dose (Section 3). Radiation dose values for questions from 2 to 9 were expressed in terms of the equivalent number of postero-anterior chest X-rays and were based on estimates from the relevant literature [3, 21, 22].

The questionnaire was administered over a period of 12 months to 780 participants equally distributed across four different main areas of Italy (i.e. North, Centre, South and Islands). Participants were asked to complete the survey within 30 min before the beginning of their course. The questionnaire was completed in the presence of an examiner and collected immediately after completion to avoid any bias. In order to prevent duplications, clear instructions were given to participants to not answer the questionnaire if they had already filled it out in previous courses or meetings. All questions of Sections 2 and 3 were in a multiple choice format with five to six options and only one correct answer. One point was given for each correct answer and zero points for each wrong or missing answer, respectively.

### Statistical analysis

Statistical analysis was performed by using software (SPSS version 17.0, www-01.ibm.com/software/analytics/spss). Categorical variables are expressed as percentages, while all continuous variables are expressed as mean ± standard deviation. The participant scores were classified by (1) their geographic distribution, (2) their level of professional experience and (3) their different levels of knowledge of radiation-related risks that were compared by using the Kruskal-Wallis test. Post-hoc analysis was performed using pairwise Mann–Whitney tests with Bonferroni correction. Questionnaire reliability was assessed as internal consistency using Cronbach’s alpha coefficient.

## Results

A total of 780 Italian radiographers completed the questionnaire. Only 12.1 % of participants claimed to be attending radiation protection training update courses on a regular basis, while 56.4 % and 31.5 % of them rarely took or had never taken any such courses, respectively. Despite this, about 90 % of the participants stated to have a sufficient knowledge of radiation protection issues.

Given a score of 1 for each correct answer and a score of 0 for incorrect or missing answers, the total mean score was 8.53 out of 16. Results were better for theoretical knowledge (average score of 4.63 out of 7) than for procedure radiation doses (average score of 3.90 out of 9). The questionnaire was found to have an acceptable internal reliability (α = 0.760; CI, 0.722–0.778).

### Radiation protection awareness

**Fig. 1:**
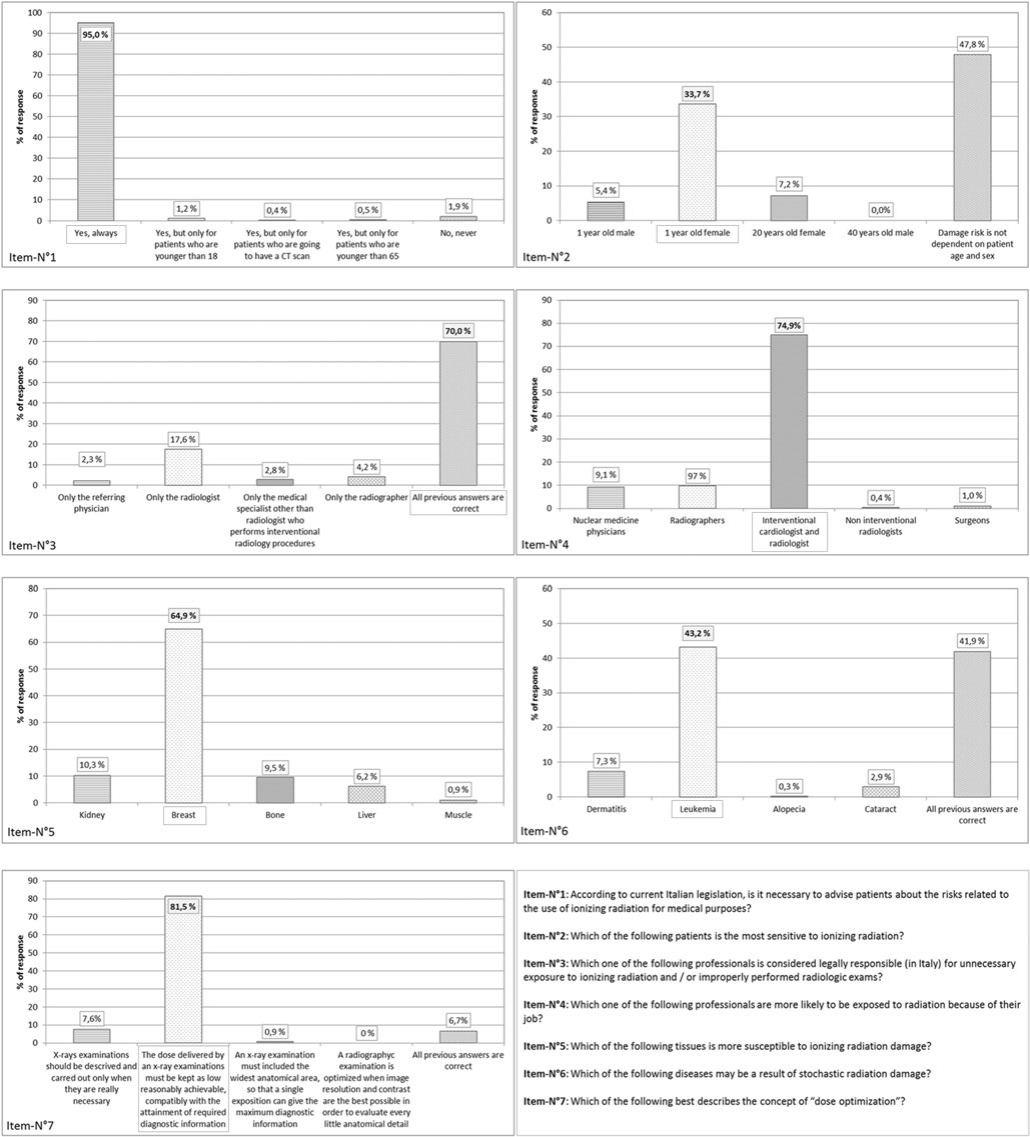
Descriptive statistical results of radiographers’ knowledge about radiation questions concerning general radiation protection issues. Right answers are *highlighted*

Concerning the questionnaire section related to general radiation protection knowledge (Fig. 1), almost all participants (95 %) showed an awareness of the need to communicate to the patient the possible risks related to radiation exposure. On the contrary, only 33.7 % of participants correctly stated that female babies are more likely to develop a radiation-induced cancer than other gender/age categories, and almost half of those respondents (47.8 %) reported that radiation risk is independent of gender and age. About 30 % of radiographers did not know that all professionals (radiologists, radiographers, referring physicians) can be legally prosecuted for the lack of appropriateness and optimisation criteria during a radiological examination. The higher radiation risk for interventional radiologists and cardiologists was correctly identified by 75 % of the participants. About one-third of radiographers (34.1 %) were not aware that the breast is the tissue with the highest susceptibility to radiation damage. Leukaemia was recognised as the result of stochastic damage by less than half of radiographers (43.2 %). Finally, 81.5 % of the respondents chose the correct definition for “dose optimisation”.

### Dose estimates for various imaging modalities

Concerning the questionnaire section in which participants were asked to assign the right dose value to natural radiation background and daily radiological procedures, 5.0 % and 3.9 % of the participants, respectively, claimed that pelvis magnetic resonance imaging (MRI) and abdominal ultrasound (US), which actually do not use ionising radiation, exposed patients to radiation. At the same time, 7.0 % of the radiographers stated that mammography does not use ionising radiation. The dose of a postero-anterior chest X-ray reported in literature amounts to about 0.02 mSv; 50.8 % of participants were able to recognise the correct dose, while 24.2 % of them overestimated it and 13.5 % of respondents reported a dose lower than 0.01 mSv. The overall distribution of answers concerning natural background radiation and commonly evaluated examination doses are shown in Table 1.

**Table 1:** Overall distribution of answers concerning the dose of natural background radiation and commonly performed examinations. Values are expressed in terms of equivalent number of PA-chest X-rays. Right answers are shown in *italics*

	Number of equivalent chest PA X-rays					
	0	1-10	10-50	50-100	100-500	>500
Italian natural background radiation	4.4	51.4	17.7	14.2	*10.3*	2.0
Lumbar spine X-ray	0.3	47.8	30.5	*16.3*	4.6	0.4
Bilateral mammography	7.0	50.6	*28.2*	10.6	3.0	0.7
(two projections each)						
Chest CT (without contrast)	1.0	2.8	16.3	26.1	*46.9*	7.0
Pelvis MRI	*95.0*	1.7	0.8	0.9	1.1	0.4
F^18^-FDG PET CT	0.7	4.8	9.4	14.3	28.6	*42.2*
Abdominal US	*96.1*	1.5	1.1	0.4	0.8	0.1
99mTc-Sestamibi cardiac scintigraphy	0.9	10.3	15.2	15.4	27.9	*30.3*
(stress + rest test, 2-day protocol)						

### Influence of geographic distribution, years of experience and knowledge awareness

Table 2 shows the level of knowledge of ionising radiation related risks, years of experience and geographic distribution for the score questionnaire, while taking into account any possible variations (in terms of score) between the different categories. The self-perception of the participants’ knowledge had no influence on the score, since there was essentially no statistical difference between the radiographers who asserted that they had a high level of knowledge and those who replied that their level of knowledge was insufficient. In fact, on the contrary, radiographers with fewer years of experience (less than 3 years) had a higher level of knowledge compared to the more experienced radiographers, with an increase in the formers’ score by about one point (Mann–Whitney tests with Bonferroni correction *p* < 0.001). No statistically significant differences were found related to the territorial distribution of the participants.

**Table 2:** Descriptive statistics and Kruskal-Wallis test of score questionnaire between the level of awareness of radiation knowledge, years of experience and geographic distribution

		Questionnaire score						
		Mean	SD	Median	IQR	Min–max	*n*	*p* value
How do you consider your knowledge level about ionising radiation related risks	Excellent	7.6	3.4	7	6–10	1–14	11	ns
	Good	8.3	2.4	8	7–10	2–14	199	
	Sufficient	8.7	2.6	9	7--11	1–16	478	
	Insufficient	8.1	3.3	8	6–11	1–14	77	
What is your level of experience	Less than 3 years	9.7	2.3	10	8–11	2–16	175	<0.001
	4-10 years	8.8	2.4	9	7–10	1–14	180	
	11-20 years	8.0	2.5	8	6–9	1–14	144	
	More than 20 years	7.9	2.7	8	6--10	1–14	250	
Regional division	North	8.9	2.2	9	8–11	5–14	150	ns
	Centre	8.2	2.5	8	7–10	2–14	184	
	South	8.6	2.8	9	6–11	1–14	175	
	Islands	7.8	2.6	8	6–10	1–13	187	

*SD* standard deviation, *IQR* interquartile range, *Min-max* minimum and maximum values, *n* number, *ns* not significant

## Discussion

To the best of our knowledge, this is the first survey carried out in Italy with the aim to evaluate knowledge of radiation protection and radiological dose assessment among Italian radiographers. Our findings from this large survey show an inaccurate awareness and training of radiographers and confirm prior studies assessing awareness of radiation protection issues and knowledge of radiation doses in different groups of specialists [8–11]. As for radiologists, radiographers’ unawareness is of particular concern as this category plays a fundamental role in the radiation protection chain. Radiographers, following the instructions given by the radiologists who must justify the procedure in advance, determine the radiation dose of the radiological examination. If the radiographer does not have an appropriate awareness of the radiation protection issues, he may be responsible for unnecessarily increasing the radiation dose delivered to the patient for a given imaging test.

Analysing the present study in detail, it is surprising that a high percentage of radiographers had rarely or never attended specific training events about radiation protection, especially considering that the Italian EURATOM 43/97 transposition imposes the attendance of at least one radiation protection course every 5 years [23]. In addition, results show no statistical difference between radiographers who claim to have an appropriate awareness about radiation protection issues and radiographers who claim to have an insufficient knowledge, outlining a lack of ability to estimate properly their own skills. On the contrary, a small but significant difference in knowledge was found depending on the level of experience; young radiographers (with less than 3 years of experience) show to have a slight increase in score when compared with older radiographers. This may be due to the fresh study course of younger radiographers (or probably, also because of the recent change of radiographers educational system). Territorial differences were not found, indicating a homogeneous knowledge level among Italian radiographers.

Almost half of respondents were not able to differentiate a stochastic effect from a deterministic effect and about 40 % of respondents assessed that radiation damage occurrence is not dependent on patient gender and age. This inaccurate knowledge raises some doubts on radiographers’ skills, which are fundamental to optimise daily radiological examinations. A poorly informed radiographer can put the patient at a higher risk by not optimising all radiation-related imaging parameters and, furthermore, might give inaccurate answers to patient questions related to the risk of the examination, as confirmed in previous studies. To this latter respect, Foley and co-workers [24] stated that a significant number of radiographers do not alter CT parameters based on either anatomical region or study indication, while another survey conducted by Briggs-Kamara and co-workers [25] showed that more than 60 % of the radiographers did not give any explanation to patients before the procedure. This lack of instruction may generate fear in patients and prevent a good cooperation during the examination, along with a higher risk of needing to repeat it [17].

The outcome of the second section of the questionnaire reveals an underestimation of doses of various radiological procedures. No one was able to complete this section without making any mistake and, surprisingly, results show that professionals of the radiological area still have doubts about which procedures make use of ionising radiation and which do not, as found in the questions related to MRI, US and scintigraphy. This result is consistent with previous studies reporting that US and MRI were associated with radiation by participants in a similar percentage to that observed in our study [26–30].

Radiation protection is the professional core of radiographers; therefore, lack of basic radiation protection awareness is unacceptable. As written in the BSS 59/13, the radiographer plays an important role representing the last gatekeeper in the radiation protection chain. Even if this lack of awareness could represent only a small risk for the individual patient, the danger becomes significant when considered at a population level. The authors suggest investigating the causes of this lack of knowledge on such fundamental topics, and then to plan actions in order to remove them. In the authors’ opinion, unawareness may depend on:

- Lack of proper preparation within university courses. Recently, Italian radiographers’ associations and Italian universities have worked in cooperation to improve teaching, but maybe further steps can be taken.
- Poor training events for staff already in employment and lack of interest in the participants, especially concerning the more senior staff.
- The increasingly difficult training caused by the growth of technological complexity, which requests the radiological staff to completely reconsider their knowledge.
- Lack of accountability, as doses are usually not collected in a unique management tool and performances are not evaluated taking dose into account.

After identifying the cause, it would be important to plan different actions to rectify this situation; e.g. by appropriate training, by creation of a multidisciplinary “dose team” and by auditing on a regular basis. Training must be performed on a regular basis by professionals with certified expertise, focusing on dosimetry concepts and optimisation measures, dose reference levels, radiation protection rules, new research studies and relevant publications. Working as a team is also an essential prerequisite to avoid wrong practices and to constantly verify the appropriateness and the optimisation of daily performed radiological examinations.

Summarising, radiographers should:

- Be provided with intensive education programs on doses per application, risk/benefit analysis and biological effects of radiation
  Attend obligatory radiation safety courses during their undergraduate studies, as well as postgraduate radiation protection and radiation safety training
- Attend updating courses about new technologies and devices which can limit radiation dose without compromising the image quality
- Be familiar with software which allows radiation dose monitoring of daily performed examinations
- Participate in projects of radiological procedures benchmarking
- Be included in multidisciplinary teams with the aim of setting up and periodically reviewing diagnostic reference levels both for adult and paediatric patients

Our work has some limitations. First of all, the questionnaire was distributed mainly during courses, meetings and workshops, so our sample refers to this specific population. Besides, the study does not take into account the data depending upon the type of radiography training, the duration of the university course and the number of years that have passed since the bachelor’s degree. Finally, there is additional information missing on the medical sector in which radiographers have worked during their careers.

In conclusion, our study shows that the knowledge of Italian radiographers about radiation protection issues and doses of radiological procedures as outlined by our survey is limited. Specific actions must be set up in order to increase awareness of radiation risks and to promote education in radiation protection with the purpose of ensuring patient safety.
