# Erratum to: Assessment of radiation protection awareness and knowledge about radiological examination doses among Italian radiographers

**DOI:** 10.1007/s13244-015-0458-1

**Published:** 2016-01-15

**Authors:** F. Paolicchi, F. Miniati, L. Bastiani, L. Faggioni, A. Ciaramella, I. Creonti, C. Sottocornola, C. Dionisi, D. Caramella

**Affiliations:** Diagnostic and Interventional Radiology, Via Roma 67, 56100 Pisa, Italy; Institute of Clinical Physiology, National Research Council, Via Moruzzi 1, 56124 Pisa, Italy; Department of Medical Physics, Piazzale Ospedale 1, 31100 Treviso, Italy

**Erratum to: Insights Imaging**

**DOI 10.1007/s13244-015-0445-6**

Unfortunately, the authors have discovered an error in the published Fig. 1. This was a transcription error; the correct percentage allocated to “Radiographers” should be 9,7 %, not 97 %.

**Fig. 1:**
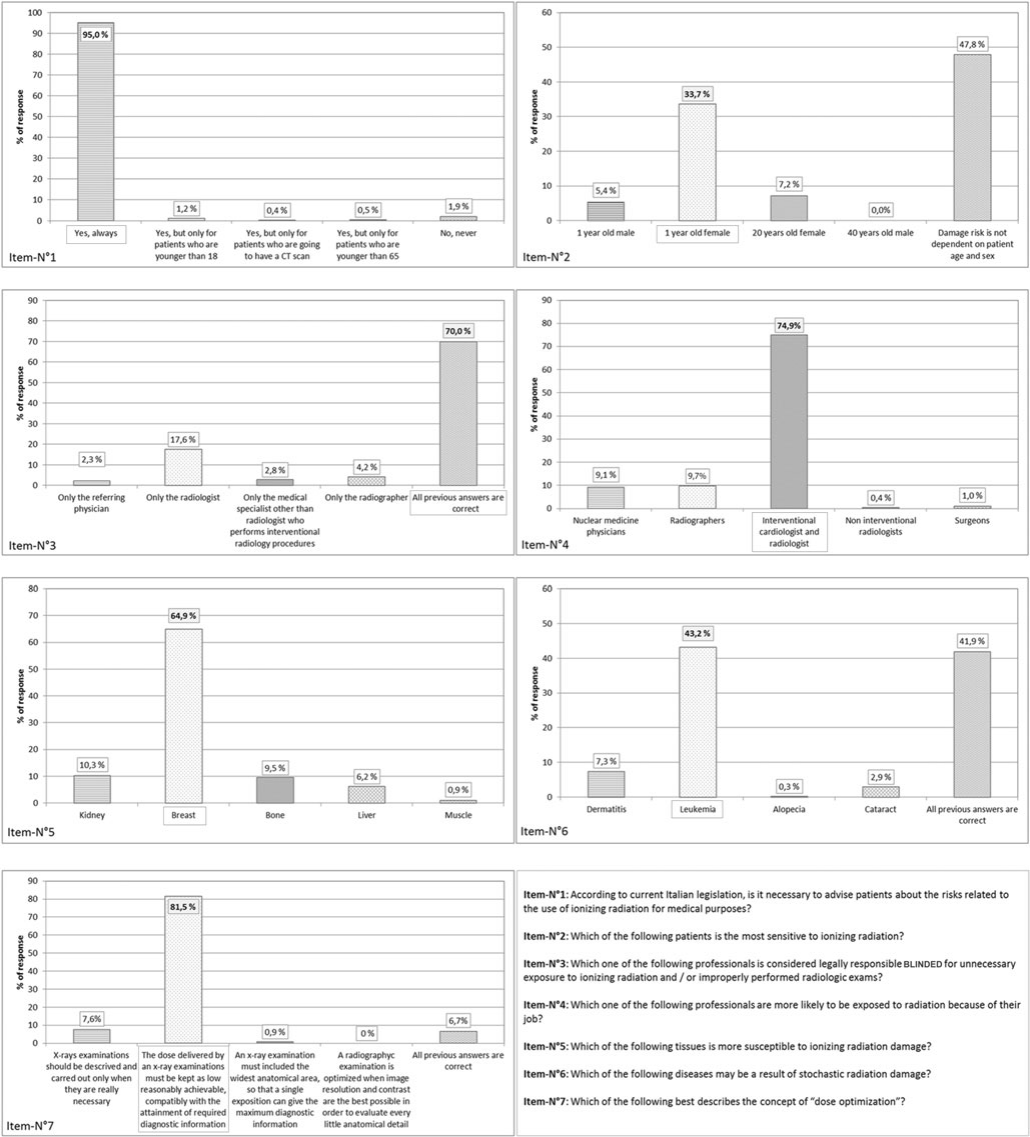
Descriptive statistical results of radiographers’ knowledge about radiation questions concerning general radiation protection issues. Right answers are *highlighted*

The authors sincerely apologize for this error.

